# Role of DJ-1 in the mechanism of pathogenesis of Parkinson's disease

**DOI:** 10.1007/s10863-019-09798-4

**Published:** 2019-05-03

**Authors:** Ludmila P. Dolgacheva, Alexey V. Berezhnov, Evgeniya I. Fedotova, Valery P. Zinchenko, Andrey Y. Abramov

**Affiliations:** 1 Institute of Cell Biophysics Russian Academy of Sciences, Pushchino, 142290 Russia; 2 Department of Clinical and Movement Neurosciences, UCL Institute of Neurology, London, WC1N 3BG UK

**Keywords:** Parkinson’s disease, DJ-1, mitochondria, neurodegeneration, oxidative stress

## Abstract

DJ-1 protein has multiple specific mechanisms to protect dopaminergic neurons against neurodegeneration in Parkinson's disease. Wild type DJ-1 can acts as oxidative stress sensor and as an antioxidant. DJ-1 exhibits the properties of molecular chaperone, protease, glyoxalase, transcriptional regulator that protects mitochondria from oxidative stress. DJ-1 increases the expression of two mitochondrial uncoupling proteins (UCP 4 and UCP5), that decrease mitochondrial membrane potential and leads to the suppression of ROS production, optimizes of a number of mitochondrial functions, and is regarded as protection for the neuronal cell survival. We discuss also the stabilizing interaction of DJ-1 with the mitochondrial Bcl-xL protein, which regulates the activity of (Inositol trisphosphate receptor) IP_3_R, prevents the cytochrome c release from mitochondria and inhibits the apoptosis activation. Upon oxidative stress DJ-1 is able to regulate various transcription factors including nuclear factor Nrf2, PI3K/PKB, and p53 signal pathways. Stress-activated transcription factor Nrf2 regulates the pathways to protect cells against oxidative stress and metabolic pathways initiating the NADPH and ATP production. DJ-1 induces the Nrf2 dissociation from its inhibitor Keap1 (Kelch-like ECH-associated protein 1), promoting Nrf2 nuclear translocation and binding to antioxidant response elements. DJ-1 is shown to be a co-activator of the transcription factor NF-kB. Under nitrosative stress, DJ-1 may regulate PI3K/PKB signaling through PTEN transnitrosylation, which leads to inhibition of phosphatase activity. DJ-1 has a complex modulating effect on the p53 pathway: one side DJ-1 directly binds to p53 to restore its transcriptional activity and on the other hand DJ-1 can stimulate deacylation and suppress p53 transcriptional activity. The ability of the DJ-1 to induce activation of different transcriptional factors and change redox balance protect neurons against aggregation of α-synuclein and oligomer-induced neurodegeneration.

## Introduction

Parkinson's disease (PD) is a neurodegenerative, multifactorial movement disorder (Zaltieri et al. 2015).

This is the age-dependent disease which affects 1% people over 60 years, growing to 4% at the age of 80 years). However, people of 18-30 years can be affected by juvenile parkinsonism, which include symptomatic parkinsonism due to brain damage (trauma, toxins, encephalitis, hypoxia) and parkinsonism developing at other neurodegenerative diseases (Huntington's chorea, dementia with Lewy bodies, multisystem atrophy). PD is clinically characterized by uncontrollable tremor at rest, rigidity, slowness of movement and postural impairment. In addition to violations of the motor function, PD is accompanied by violations of the gastrointestinal, olfactory, sleep, cognitive and other disorders. These symptoms are the result of loss of function and/or death of the majority of dopaminergic neurons of the midbrain with subsequent disruption of dopaminergic neurotransmission in the dorsal striatum where the presynaptic endings of these neurons are located. PD is characterized by progressive death of midbrain dopaminergic neurons of substantia nigra pars compacta (SNc) (Barzilai and Melamed 2003) and the presence of intracellular inclusions called Lewy bodies consisting mainly of aggregated α-synuclein (α-Syn) (Braak et al. 1999; Trojanowski and Lee 1998). The sporadic form of PD is associated with various environmental factors, including the effects of neurotoxins (MPTP), pesticides and herbicides such as rotenone and paraquat (Betarbet et al. 2000; Jenner 2003; Przedborski et al. 2004). Hereditary forms caused by mutations in several genes constitute 10-15% of all cases of PD (Sherer et al. 2002). Currently, more than 15 genes of Parkinsonism hereditary forms have been identified. Mutations in the genes LRRK2 (enriched with leucine repeats kinase 2) and SNCA (alpha-sinuclein) are well-known causes of autosomal dominant Parkinson's disease, mutations in the Parkin, PINK1 and DJ-1 genes, mediate autosomal recessive and early forms of PD (Bonifati et al. 2003a; Annesi et al. 2005).

Mutations in these genes are strongly associated with mitochondrial dysfunction and oxidative stress (Bonifati et al. 2003b; Valente et al. 2004; Abramov et al. 2017). Axons of the nigrostriatal system form one of the longest tracts in the brain and require an additional ATP to transport the components to the distally located synaptic terminals (Braak et al. 2004; Fu et al. 2016). A deficit of ATP and mitochondrial Ca^2+^ overload can be a trigger for neurodegenerative diseases (Abeti and Abramov 2015; Ludtmann and Abramov 2018).

DJ-1 protein plays a role of oxidative stress sensor - it eliminates peroxide by autoxidation (Mitsumoto and Nakagawa 2001). DJ-1 is also strongly implicated in pathogenesis of cancer and suggested to be one of potential tumor marker (Yu et al. 2017; Fan et al. 2016).

**Fig. 1:**
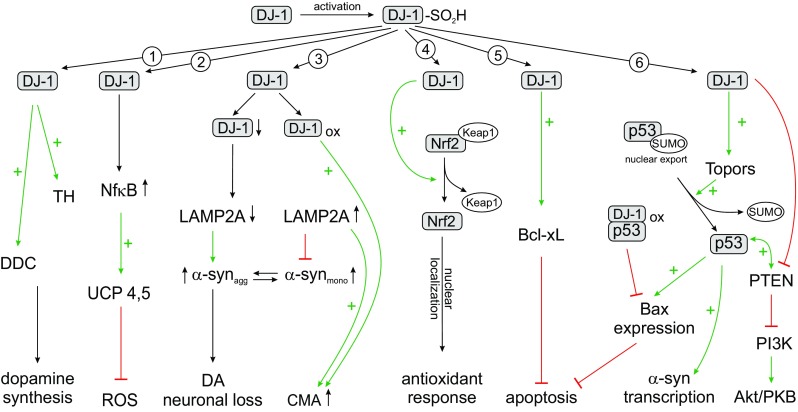
Diversity of the effects of DJ-1 in cell. 1. DJ-1 is able to upregulate dopamine synthesis via direct activation of tyrosine hydroxylase (TH) and 4-dihydroxy-L-phenylalanine decarboxylase (DDC). 2. In the nucleus DJ-1 acts as a transcriptional coactivator of NF-kB and subsequent transcription of the gene encoding UCP4. UCP-induced mild uncoupling can reduce the ROS production. 3. DJ-1 prevents potentially toxic a-syn aggregation via activation of a-syn degradation by the chaperone-mediated authophagy (CMA). 4. DJ-1 stimulates endogenous antioxidant system by the activation of Nrf2. 5. DJ-1 upregulates and stabilizes Bcl-xL in mitochondria preventing apoptotis. 6. DJ-1 positively regulates p53 through Topors-mediated sumoylation. Overexpression of DJ-1 decreases the expression of Bax and inhibits apoptosis. DJ-1 also inhibits PTEN to activate PI3K/PKB (Akt) pathway

DJ-1 participates in a number of signaling pathways, including control of mitochondrial quality and reaction to oxidative stress. It has been shown that cells with a high level of DJ-1 are resistant to both oxidative stress and to neurotoxins such as 6-OHDA (6-hydroxydopamine), while lower levels of DJ-1 make cells to be vulnerable to oxidative stress (Fig. 1) (Taira et al. 2004; Inden et al. 2011).

**Fig. 2:**
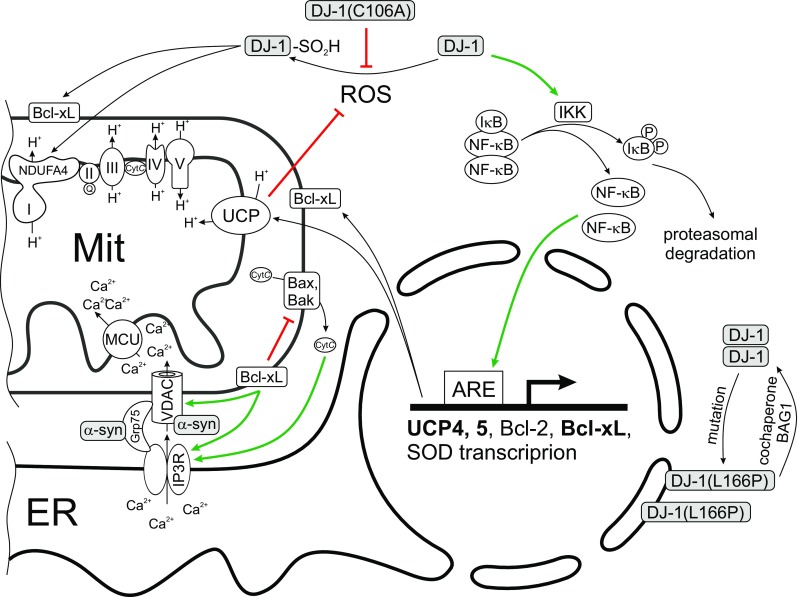
ROS activated DJ-1 is able to interact with complex I and maintain its activity. In addition, DJ-1 suppresses ROS overproduction, triggering expression of the gene encoding UCP. This process is mediated by activation of IκB kinase followed by activation of the transcription factor NF-κB and expression of genes encoding UCP4, UCP5 and Bcl-xL. UCP causes a mild uncoupling of oxidative phosphorylation, suppressing the production of ROS and thereby regulating the level of ROS on the principle of negative feedback. Bcl-xL is able to control mitochondrial and reticular Ca^2+^ transport through the activation of IP3R and VDAC – the components of the MAM complex. The main role of Bcl-xL is to suppress the apoptosis. Mutations in the gene encoding DJ-1 lead to disruption of these functions. So replacing the C106A blocks the activation of DJ-1 by reactive oxygen species, and the L166P mutation provides the nuclear localization of DJ-1

Knockout of the DJ-1 gene reduced the expression of two mitochondrial uncoupling proteins (UCP4 and UCP5 – see Figs. 1 and 2), impaired the function of calcium-induced uncoupling and increased the oxidation of matrix proteins in substantia nigra pars compacta (SNc) dopaminergic neurons (Surmeier et al. 2010). Recent studies have shown that DJ-1 protects dopaminergic neurons against oxidative damage not only *in vitro*, but also *in vivo* (Bjorkblom et al. 2013; Choi et al. 2014; Mullett et al. 2013; Tanti and Goswami 2014). Oxidised DJ-1 was shown to be significantly decreased in idiopathic PD brain, suggesting altered complex function controlled by DJ-1 may also play a role in the more common sporadic form of the disease (Piston et al. 2017).

This review summarise neuroprotective role of DJ-1 through regulation of α-Syn quality control, chaperone-mediated autophagy, antioxidant protection of neurons, oxidative phosphorylation, anti-apoptotic effect of Bcl-xL and the regulation of signalling pathways in the context of PD.

## Structure, functions and mechanism of DJ-1 action

The DJ-1 gene was first discovered as a new mitogen-dependent oncogene involved in the Ras-dependent signal transduction pathway (Nagakubo et al. 1997). DJ-1 is a 24 Kb gene that encodes a protein with 189 amino acid residues (Moore et al. 2006; Moore et al. 2005; Trempe and Fon 2013). It is a small ubiquitously expressed protein with a molecular mass of about 20 kDa (Bader et al. 2005). The crystal structure of this protein was investigated by several independent research groups (Honbou et al. 2003; Huai et al. 2003; Tao and Tong 2003; Wilson et al. 2003). The protein exists as a homodimer in the cytoplasm, mitochondria, and nucleus (Zhang et al. 2005). DJ-1 is a protein sensor that reacts to oxidative stress and protects cells from ROS (Taira et al. 2004; Inden et al. 2006). DJ-1 has been shown to function as a dimer and contains an essential cysteine residue within its active site that functions as an oxidative sensor. Studies have shown that the brains of patients with Alzheimer's disease and Parkinson's disease contain a high level of oxidized DJ-1, which is believed to possess neuroprotective properties (Choi et al. 2006; Bandopadhyay et al. 2004). DJ-1 has three cysteine residues in its amino acid sequence at residues 46, 53 and 106 in humans and rats. It was shown that the cysteine residue C106 in DJ-1 is the most sensitive site to oxidation by hydrogen peroxide (H_2_O_2_) (Kinumi et al. 2004). Of the three cysteine residues, the oxidative status of the amino acid cysteine residue C106 determines the active level of the DJ-1 protein. Cys-106 of DJ-1 is sequentially oxidized from the reduced form (-SH) to sulfenated form (-SOH), sulfinated form (-SO_2_H), and sulfonic form (-SO_3_H). The degree of oxidation at the C106 residue determines DJ-1 activity (Choi et al. 2014; Ito et al. 2006; Wilson 2011). Thus, active form of DJ-1 is with sulfinated C106, sulfonic form of C106 in DJ-1 is inactivating this peptide. Inactive SO_3_H form of DJ-1 found in patients with sporadic PD suggesting that DJ-1 can be involved not only familial but also in sporadic PD (Ariga et al. 2013). In addition to performing the sensory function of oxidative stress, DJ-1 neutralizes reactive oxygen species (ROS) (Taira et al. 2004; Cookson 2003), is a molecular chaperone (Meulener et al. 2005; Shendelman et al. 2004), protease (Chen et al. 2010), glyoxalase (Lee et al. 2003), the transcriptional regulator, the RNA-binding protein, the mitochondrial function regulator and the autophagy regulator (Trempe and Fon 2013; Ariga et al. 2013; Richarme and Dairou 2017). DJ-1 shown to be a redox sensitive adapter protein for high molecular weight complexes involved in regulation of catecholamine homeostasis, specifically noradrenaline and dopamine (Fig. 1) (Piston et al. 2017).

## Deficiency of DJ-1

DJ-1 is a multifunctional protein and mutations in its gene are associated with a number of diseases such as neurodegenerative diseases, stroke, type II diabetes and cancer (Choi et al. 2006; Ariga et al. 2013; Kahle et al. 2009; Aleyasin et al. 2007; Inberg and Linial 2010; Cao et al. 2015; Jain et al. 2012). The homozygous deletion or point mutation in the human DJ-1 gene that lead to the replacement of the proline amino acid residue by leucine (L166P) causes an autosomal recessive early form of PD (Bonifati et al. 2003b). The crystal structures of wild DJ-1 and mutated L166P proteins demonstrate that the L166P mutation prevents normal folding of the C-terminal region. Such a change in the structure leads to a disruption in transport properties and the ability to form dimers. In contrast to DJ-1 which forms soluble dimers, the mutant L166P exists in cells as a monomer (Moore et al. 2003; Anderson and Daggett 2008), and loses its physiological functions and acquires proapoptotic properties (Ren et al. 2012). The PD-associated loss of DJ-1 function is related to reduce lysosomal activity and mitochondrial damage (Krebiehl et al. 2010). DJ-1 activity is abrogated by the Park7 (L166P) mutation, associated with primary parkinsonism (Shendelman et al. 2004).

## DJ-1 in α-Synuclein aggregation and quality control

Misfolding and oligomerisation of α-Synuclein (α-Syn) involved in both hereditary and sporadic forms of Parkinson's disease (Abramov et al. 2017). Autosomal familial dominant forms of PD is induced by mutations (A53T, A30P and E46K) (Polymeropoulos et al. 1997; Kruger et al. 1998; Zarranz et al. 2004), or multiplication (duplications, triplications or overexpression) (Chartier-Harlin et al. 2004) in the SNCA gene, probability of sporadic PD also may be increased by polymorphisms at the SNCA locus (Simon-Sanchez et al. 2009). Removal of misfolded protein plays a critical role in the aggregation of α-Syn and the pathogenesis of PD. Previously it was shown that DJ-1 indirectly (without co-localizations) inhibits aggregation of α-synuclein. It was suggested that DJ-1 is activated in an oxidative cytoplasmic environment and acts as a redox-sensitive molecular chaperone (Shendelman et al. 2004). DJ-1 can inhibit starting point of aggregation of α-synuclein (monomers) but not oligomerization of fibril formation (Martinat et al. 2004). Later it was found that oxidation of Cys106 to the sulfinic acid had minimal effect on the structural properties of DJ-1, whereas the SO_2_H form of C106 was very effective in preventing the fibrillation of α-Syn. Further oxidation of DJ-1 led to the loss of some secondary structure, and of the ability to inhibit alpha-synuclein fibrillation. The authors concluded that this can be a mechanism of action of DJ-1 as an oxidative-stress-induced chaperone to prevent α-Syn fibrillation (Zhou et al. 2006). More recent, it was found that DJ-1 interacts directly with α-Syn monomers and oligomers not only in vitro systems but also in living cells and mutations in DJ-1 (Park7) gene associated with PD limit this interaction. Moreover, excessive expression of DJ-1 reduced dimerization of α-Syn (Zondler et al. 2014). It was confirmed that suppression of DJ-1 (knockout)increased level of aggregated α-synuclein in cellular (SH-SY5Y) and animal models of PD, opposite - over-expression of DJ-1 *in vitro* effectively decreased α-Syn levels (Xu et al. 2017). The important neuronal physiological role of α-Syn and the central role in the pathogenesis of PD suggest a high level of correctness between the processes of synthesis and degradation of this protein. In the processes of degradation of pathogenic α-Syn prone to aggregation, a critical role is assigned to the lysosomal system (Fig. 1) (Moors et al. 2016). On the mechanisms of cytoplasmic substrates delivery to the lysosome, the autophagy-lysosome pathway can be divided into macroautophagy, microautophagy and chaperone-mediated autophagy (Cuervo and Wong 2014).

## DJ-1 regulate chaperone-mediated autophagy

Soluble wild-type α-synuclein is mainly degraded by chaperone-mediated autophagy (CMA), and impairment of CMA is closely related to the pathogenesis of PD (Xu et al. 2017; Vogiatzi et al. 2008). Under normal conditions, CMA occurs constitutively at low levels. In conditions of oxidative stress and presence of α-Syn aggregates the affinity between Hsc70 and α-synuclein fibrils is 5-fold tighter compared with soluble α-Syn (Pemberton et al. 2011).

It was shown that oxidized DJ-1 with the SO_2_H form of C106 was the active form for realization of chaperone activity (Zhou et al. 2006). Wild-type α -Syn is a substrate of CMA and CMA dysfunction may contribute an increase in pathological α -synuclein aggregates (Vogiatzi et al. 2008; Cuervo et al. 2004). Chaperone protein HSPA8 directly binds CMA substrate proteins and targets them to the lysosomes for LAMP2A-mediated degradation (Majeski and Dice 2004). CMA is a highly specific process in which cytosolic protein substrates with the KFERQ-targeting motif are recognized by Hsc70 (heat-shock cognate protein of 70 kDa) of chaperone-complex (Kaushik and Cuervo 2009; Velseboer et al. 2011). Substrate can be translocated to lysosomes by the lysosomal-associated membrane protein 2a (LAMP2A) receptor (Dice 2007; Yang et al. 2009) and then quickly degraded by the proteases (Fig. 1) (Bejarano and Cuervo 2010). Over-expression of LAMP2A in human SH-SY5Y cells or rat primary cortical neurons *in vitro* and nigral dopaminergic neurons *in vivo* decreased α-Syn accumulation and protected against α-Syn-induced dopaminergic degeneration (Xilouri et al. 2013). DJ-1 deficiency accelerated the degradation of LAMP2A in lysosomes, leading to the aggregation of a-syn. Lower levels of the CMA markers LAMP2A and the chaperone Hsc70 was observed in various regions of postmortem brain specimens from PD patients in SNc compared to controls (Alvarez-Erviti et al. 2010; Murphy et al. 2015). More recent studies confirm that DJ-1 deficiency accelerated the degradation of LAMP2A in lysosomes, leading to the aggregation of α-Syn (Xu et al. 2017). Thus DJ-1 could inhibit α -synuclein accumulation and aggregation by regulating CMA.

## The role of DJ-1 in neuronal antioxidant defense

Neurons are postmitotic cells and they characterized by high oxygen consumption, lipid content and metabolic activity that makes them more sensitive to oxidative damage compared to other cells (Gandhi and Abramov 2012; Angelova and Abramov 2016). Oxidative stress has been shown to play important roles in the pathogenesis of PD (Chien et al. 2013). DJ-1 plays a significant role in antioxidant protection of neurons against oxidative stress, acts as a sensor of oxidative stress, by interaction with other enzymes of the antioxidant system and by elevating of expression of the corresponding antioxidant defense genes (Taira et al. 2004; Inden et al. 2006; Wilson 2011; Ariga et al. 2013; Kahle et al. 2009; Liu et al. 2008; Bonifati 2012). The antioxidant defense enzyme network consists from superoxide dismutase, glutathione peroxidase, catalase and paraoxonase (Gandhi and Abramov 2012; Parsanejad et al. 2014). DJ-1 over-expression promotes an increase in the glutathione level that protects neurons from the oxidative stress caused by H_2_O_2_ and 6-OHDA (Zhou and Freed 2005). Furthermore, cells with a high level of DJ-1 are resistant to oxidative stress and neurotoxins, such as 6-OHDA, while lower levels of DJ-1 make cells vulnerable to oxidative stress (Taira et al. 2004; Inden et al. 2011; Gunjima et al. 2014). Under condition of oxidative stress, a conserved cysteine residue in DJ-1 (Cys106) is oxidized and this oxidative modification is enabling DJ-1 to act as scavenges ROS and as a sensor of cellular redox homeostasis. DJ-1 is mainly localized in the cytoplasm, but under oxidative stress it can be translocated to the mitochondria and nucleus (Irrcher et al. 2010; Kim et al. 2012) for 3 and 12 hours, respectively, acting as a cytoprotector (Fig. 2) (Junn et al. 2009; Blackinton et al. 2009). Mild oxidation of Cys106 to the sulfin form (SO2H) is necessary for mitochondrial localization and protection of cells against oxidative stress (Blackinton et al. 2009) and for inhibition of α-Syn fibrils formation (Fig. 2) (Zhou et al. 2006). DJ-1 translocated into the nucleus act as a transcriptional coactivator of NF-kB transcription factors because it does not have a separate DNA-binding site (Kim et al. 2012; Yamaguchi et al. 2012).

## Interaction of DJ-1 and mitochondria

Mitochondrial dysfunction plays a central role in the mechanism of neurodegeneration in PD. Mitochondria considered being one of the main ROS producers within the cell. ROS overproduction induces rapid relocation of DJ-1 to mitochondria, suggesting that mitochondria could be a site for DJ-1’s neuroprotective activity (Junn et al. 2009; Canet-Aviles et al. 2004). Using the electron microscopy, DJ-1 protein was identified both in the mitochondrial matrix and in the intermembrane space (Zhang et al. 2005). It was shown that DJ-1 is co-localized with the subunit of NDUFA4 mitochondrial complex I even in the absence of stress. Binding of DJ-1 to the subunits of complex I was enhanced by oxidative stress (Hayashi et al. 2009). Knockout of the DJ-1 gene in human dopaminergic neurons led to depolarization of mitochondria, their fragmentation and accumulation of autophagy markers around the mitochondria. Apparently, these effects are due to endogenous oxidative stress, since antioxidants have abrogated them. DJ-1 suppresses mitochondrial fragmentation caused by the mitochondrial toxin rotenone in the same way as PINK1 (Thomas et al. 2011). Death of the dopaminergic neurons in the SNc characterizes PD and it is the main cause of motor impairment (Fahn 2003). The axons of the nigrostriatal system form one of the longest tracts in the brain and, possibly require an additional ATP to transport the components to the distally located synaptic terminals (Braak et al. 2004; Fu et al. 2016). It was shown that a higher basal rate of mitochondrial oxidative phosphorylation and an elevated level of basal ROS production characterized nigral dopaminergic neurons compared to dopaminergic neurons of the VTA (ventral tegmental area) (Pacelli et al. 2015). Rhythmic pacemaker activity of the SNc dopaminergic neurons was suggested to be one of the reasons for high energy demand and vulnerability of these cells. SNc dopaminergic neurons are autonomous pacemakers, generating action potentials at a relatively slow rate (2-10 Hz) in the absence of synaptic input (Grace and Bunney 1983a; Grace and Bunney 1983b; Chan et al. 2007; Guzman et al. 2009; Surmeier et al. 2017). The rhythmic pacemaker activity is due to the properties of the pore-forming subunit of Cav1.3 of the L-type Ca^2+^-channels that regulate the basal level of dopamine in the striatum (Guzman et al. 2009; Kang et al. 2012; Surmeier and Schumacker 2013). Cytosolic Ca^2+^ oscillations in dopaminergic neurons of SNc initiate Ca^2+^-entry into mitochondria and stimulate ATP production (Surmeier and Schumacker 2013; Denton 2009; Aumann et al. 2011). Activation of mitochondrial respiration in the absence of high ATP demand leads to mitochondrial hyperpolarization and increased production of ROS (Pacelli et al. 2015; Guzman et al. 2010).

## DJ-1 increases the transcriptional activity of UCP4 and mild uncoupling interacting with NF-κB and stabilizes Bcl-xL

The ability of mitochondria to produce ROS (reactive oxygen species) in the electron transport chain (TCA) cycle and some other enzymes has a functional implication in cell signaling. In low level, ROS perform signaling functions as secondary intermediaries in redox-sensitive signaling pathways (Petry et al. 2010). Mitochondria produced low level of ROS even in resting conditions as an electron leakage in electron transport chain. However, production of ROS in mitochondria may be dramatically increased in case of inhibition of mitochondrial complexes which induce a reverse flux of electrons, or increase the electron leakage in case of mitochondrial hyperpolarization. Increased production of ROS induces oxidative damage of the DNA, especially mtDNA, proteins, lipids, causing oxidative stress. It is become widely accepted that oxidative stress and mitochondrial dysfunction contribute to the loss of dopaminergic neurons, age-related pathology and neurodegenerative disorders (Angelova and Abramov 2016; Surmeier et al. 2017; Balaban et al. 2005; Schapira and Jenner 2011; Zhu and Chu 2010). The rate of ROS formation in mitochondria can be decrease by using low doses of protonophores that partially reduce mitochondrial membrane potential and induces process termed “mild uncoupling” (Adam-Vizi and Chinopoulos 2006; Starkov 2008). There is a family protein on the inner mitochondrial membrane called the uncoupling proteins (UCPs), act to promote this proton leakage for thermoregulation and to prevent excessive ROS production. One of the isoforms UCPs - UCP4 is predominantly expressed in brain tissue, mostly in all brain regions including SNc and striatum (Ramsden et al. 2012). NF-κB regulates The transcriptional activity of UCP4 in both ways – it can be increased by agonist NF-kB or decreased by inhibitors of NF-kB (Ho et al. 2010). One of the important properties of NF-kB is its ability to protect the cell from apoptosis (Hoffmann and Baltimore 2006).

UCP4 knockdown decreased cellular antioxidative capacity and depolarized mitochondrial membrane under normal culture conditions (Ho et al. 2012a). In this way UCP4 expression is significantly regulated through the activation of NF-κB signaling by the NF-κB-response element binding site within the promoter region of UCP4 (Ho et al. 2012b; Xu et al. 2018). DJ-1 can increase expression of UCP4 via NF-κB pathway because DJ-1 enhances NF-κB nuclear translocation and cell survival (Xu et al. 2018). Knocking out DJ-1 downregulated UCP4 and UCP5 expression and increased oxidation of matrix proteins specifically in SNc dopaminergic neurons (Ho et al. 2012a).

Intracellular ROS are a key factor that can regulate the phosphorylation of nuclear factor IκB-α and activate NF-κB (Kretz-Remy et al. 1996; Schreck et al. 1991; Schreck and Baeuerle 1991; Asghar et al. 2006; Fardoun et al. 2007). c-Rel subunit was involved in regulating UCP4 gene expression via the NF-κB response element in the UCP4 gene promoter. c-Rel over-expression induced NF-κB activity . Overexpression of c-Rel increased UCP4 promoter activity and protein expression. Hydrogen peroxide increased NF-κB binding to the UCP4 promoter.

DJ-1 is present mostly in the cytoplasm and to a lesser extent in mitochondria and nucleus of dopaminergic neurons. It was shown that DJ-1 is translocated to the mitochondria and nucleus in response to oxidative stress (Junn et al. 2009; Canet-Aviles et al. 2004) increasing the interaction between DJ-1 and Bcl-xL (Ren et al. 2011). Anti-apoptotic Bcl-xL encoded by the BCL2-like 1 gene, is a transmembrane molecule in the mitochondria. It is a member of the Bcl-2 family of proteins, and acts as an anti-apoptotic protein by preventing the release of mitochondrial cytochrome c (Adams and Cory 1998), which leads to caspase activation. Bcl-xL forms heterodimers with pro-apoptotic Bcl-2 family proteins to inhibit apoptosis (Petros et al. 2000). As a mitochondrial anti-apoptotic protein Bcl-xL prevents oligomerization of pro-apoptotic Bax (Bcl2-Associated X protein) and Bak (Bcl-2 homologous antagonist/killer) (Cheng et al. 1996; Sattler et al. 1997; Kharbanda et al. 1997).

Previous studies have demonstrated that Bcl-xL binds to all three IP_3_R isoforms to increase their sensitivity to stimulation (Li et al. 2007). Bcl-xL has been shown to regulate mitochondrial fusion, fission, and biomass (Berman et al. 2009). Interruption of the processes of mitochondrial fission and fusion is associated with the suppression of energy and can lead to the activation of cell death mechanisms (Gellerich et al. 2009; Mandemakers et al. 2007; Seppet et al. 2004).

Under oxidative stress, Bcl-xL is degraded by the ubiquitin-proteasome system (UPS). The interaction of Bcl-xL with DJ-1 stabilizes protein level by inhibiting the ubiquitination-dependent degradation of Bcl-xL. The loss of DJ-1 leads to the mitochondrial depolarization, fragmentation and accumulation of markers of autophagy around mitochondria in human dopaminergic cells (Thomas et al. 2011). In recent studies using NMR spectroscopy was shown that the oxidized but not reduced form of DJ-1 binds to a hydrophobic groove consisting of the domains BH1-BH3 in Bcl-xL. Based on the improved structural model of the complex, the authors concluded that the interaction of the C-terminal α-helical region of DJ-1 with Bcl-xL is similar to interaction of pro-apoptotic domains of BH3 with Bcl-xL (Lee et al. 2018). It should be noted that Bcl-xL interacts with F_1_F_0_ ATP synthase and optimizes the function by enhancing mitochondrial ATP production and decreasing proton leakage across the mitochondrial inner membrane (Alavian et al. 2011; Chen et al. 2011). Thus, one of the important protective roles of DJ-1 in oxidative stress is mediated by the stabilization of Bcl-xL in mitochondria (Lee et al. 2018).

## DJ-1 and the regulation of signaling pathways

DJ-1 has been suggested to orchestrate different cellular pathways involved in response to oxidative stress. DJ-1 expression is upregulated under oxidative stress conditions and protein translocate into the nucleus upon exposure to stress, suggesting a key role in gene transcription (Kim et al. 2012). It should be noted that DJ-1 does not exhibit DNA-binding domain suggesting that it acts as a co-activator of transcription (Yamaguchi et al. 2012). DJ-1 regulates gene expression and mitochondrial integrity, induces survival pathways and protein refolding (Raninga et al. 2017). Upon oxidative stress, DJ-1 has been shown to regulate various transcription factors including nuclear factor Nrf2, p53 and PI3K/PKB signaling cascade that transmit downstream signals to respond to oxidative stress (Shinbo et al. 2005; Clements et al. 2006).

## p53 pathway and DJ-1

p53 protein is a transcription factor which can induce transcription of several genes encoding proteins involved in a wide spectrum of biochemical processes including DNA repair, cell-cycle regulation, and programmed cell death. Activation of p53 is induced by a number of stress signals, including DNA damage, oxidative stress and activated oncogenes (Horn and Vousden 2007; el-Deiry et al. 1992; Funk et al. 1992; Beckerman and Prives 2010). Activity of p53 is also provided by a number of modifications that affect the localization of p53: phosphorylation, acetylation, ubiquitination, methylation and sumoylation (Gu and Zhu 2012). DJ-1 can do both - regulates and to be regulated by p53 (Giaime et al. 2010; Duplan et al. 2013).

Association between DJ-1 and p53 is dependent on redox state of DJ-1’s C106 residue (Kato et al. 2013). Oxidative status of a cysteine residue at position 106 (C106) is crucial for determination of the activation level of DJ-1.

SUMOylation (SUMO - small ubiquitin-like modifier proteins) of proteins outside the nucleus plays direct roles in controlling synaptic transmission, neuronal excitability, and adaptive responses to cell stress (Henley et al. 2018). It was shown that DJ-1 positively regulates p53 through Topors-mediated sumoylation (Shinbo et al. 2005). Topors (a ring finger protein binding to both topoisomerase I and p53) was originally identified as cellular binding partner of DNA topoisomerase I and of p53 with function similar to ubiquitin E3 ligase for p53 (Rajendra et al. 2004). DJ-1 directly binds to p53 that inhibit sumoylation of p53 through interaction of DJ-1 with Topors. DJ-1 protein affects the tumor suppressor p53 Bax-caspase pathway, which triggers apoptosis. It induces cell survival in a redox-dependent manner by decreasing the apoptosis regulator Bax (BCL2-Associated X protein) expression (Fan et al. 2008). DJ-1 protects neurons against caspase activation and cell death via expression/ suppression of Bax (Bretaud et al. 2007).

α-synuclein was also identified as a new transcriptional target of p53. Duplan E, et al showed that p53 interacts physically with α-synuclein promoter. Deletion of p53 responsive element from α-Syn promoter abrogates p53-mediated α-Syn regulation (Duplan et al. 2016).

It was shown that DJ-1 stimulates deacetylase activity of SIRT1, which deacetylate p53, suppresses its transcriptional activity and prevents apoptosis. In addition, it was shown that the activity of SIRT1 was reduced in the cells with DJ-1 knockout, and the activity level was restored as a result of repeated addition of wild-type DJ-1 (Takahashi-Niki et al. 2016).

## DJ-1 inhibits PTEN to activate PI3K/PKB (Akt) pathway

The PI3K/PKB signaling cascade plays critical role in regulating diverse cellular functions including metabolism, growth, proliferation, survival, protein synthesis that lead to protection in number of neurodegenerative disorders. Under nitrosative stress, DJ-1 regulate PI3K/PKB signaling by transnitrosylation. NO nitrosylates PTEN and inhibits its phosphatase activity (Numajiri et al. 2011). Later transnitrosylated PTEN (SNO-PTEN) was detected in human brains with sporadic PD. DJ-1 could also be *S*-nitrosylated by endogenous NO generated from neuronal nitric oxide synthase 1 (nNOS) (Choi et al. 2014). It was shown that PTEN and DJ-1 form a complex in cells and under mild nitrosative stress, inhibition of PTEN activity in this case is produced via providing NO group through SNO-DJ-1, with subsequent transnitrosylation to form SNO-PTEN (Choi et al. 2014).

## DJ-1 stabilizes Nrf2 that prevent binding to Keap1

Nrf2 is an environmental and stress-activated transcription factor which upregulates the expression and activity enzymes involved in defense toxic and oxidative stress and increase energy production in form of NADPH, NADH in the metabolic pathways (Holmstrom et al. 2013; Esteras et al. 2016). DJ-1 may be involved in control Nrf2 activity though inhibitory protein Keap1 under oxidative stress.

One of the major and most intensive ROS producer in the cell, enzyme NADPH oxidase, which originally identified in phagocytes, recently shown to be expressed in most of the tissues including brain cells (Droge 2002; Bedard and Krause 2007). Two isoforms NADPH oxidase NOX2 and NOX4 expressed in neurons and glia (including microglia and astrocytes) (Bedard and Krause 2007; Park et al. 2008; Dohi et al. 2010; Abramov and Duchen 2005). Activation or inhibition of Nrf2 (by Keap1 or Nrf2 deficiency) lead to opposite effect for NOX2 and NOX4, mRNA expression of NOX2 rose in inhibited Nrf2 while NOX4 in (Kovac et al. 2015; Dinkova-Kostova et al. 2015). There are increasing number of reports suggesting involvement of Nrf2 in normal ageing and neurodegenerative diseases. Pharmacological activation of Nrf2 and some substrates (nicotinamide)shown to be protective against neuronal damage (Ghosh et al. 2014). Besides effect of Nrf2 activity on expression and activity of NADPH oxidase, both activation and inhibition of Nrf2 lead to increased mitochondrial ROS production (Kovac et al. 2015). DJ-1 activates Nrf2 by dissociation with Keap1, promoting Nrf2 binds with and activates specific antioxidant and detoxifying gene expression (Fig. 1). Nrf2 is shown to be responsible for transcription of the antioxidant proteins: gluthatione S-transferases (GSTs), NAD(P)H: quinone oxidoreductase 1, thioredoxin, thioredoxin reductase (Motohashi and Yamamoto 2004; Kensler et al. 2007; Im et al. 2012; Gorrini et al. 2013). In the production thioredoxin1 DJ-1 act through Nrf2–antioxidant responsive element (ARE) pathway rather than through Nrf2 and Keap1 interaction. Active residues of DJ-1 involved in increase of thioredoxin1 expression were identified by absence of this activity in mutations in M26I and L166P and a missense mutant at C106S (Im et al. 2012; Im et al. 2010).

Evidence of the effect of DJ-1 on the stability and transcriptional function of Nrf2 was obtained using both as cell lines as animals. It was found that DJ-1 stabilizes Nrf2, preventing its ubiquitination, preventing binding to Keap1 and facilitating the translocation of Nrf2 into the nucleus (Clements et al. 2006; Gan et al. 2010). In the absence of DJ-1, Nrf2 is unstable and the activity of antioxidant enzymes is lower due to inhibition of the Nrf2 pathway (Taira et al. 2004; van Horssen et al. 2010; Lee et al. 2012). ROS production is dramatically increased in brain tissue from Nrf2-KD mice and the Keap1-Nrf2 pathway plays a key role in redox homeostasis within the cell (Kovac et al. 2015). DJ-1 is involved in the Nrf2-dependent oxidative stress response that leads to the upregulation of both the 20S proteasome and its regulator, NQO1 (NAD(P)H quinone dehydrogenase 1) (Moscovitz et al. 2015). The authors demonstrated that DJ-1 physically binds the 20S proteasome and inhibits its activity, rescuing partially unfolded proteins from degradation. DJ-1 is involved in the upregulation of Nrf2, the 20S proteasome regulator NQO1, the 20S proteasome and sustains the balance between the need to rapidly eliminate oxidatively damaged proteins and maintain the abundance of native, intrinsically unstructured proteins, which coordinate regulatory and signalling events (Moscovitz et al. 2015).

In the conclusion DJ-1 has multiple specific mechanisms for protecting dopamine neurons from cell death in Parkinson's disease. DJ-1 plays the role of oxidative stress sensor and antioxidant. DJ-1 exhibits the properties of molecular chaperone, protease, glyoxalase, transcriptional regulator and protects mitochondria from oxidative stress. DJ-1 effects the α-synuclein aggregation. DJ-1 increases the expression of mitochondrial uncoupling proteins which leads to the suppression of ROS production. Stabilizing interaction of DJ-1 with the mitochondrial Bcl-xL prevents the cytochrome c release from mitochondria and inhibits the apoptosis activation. DJ-1 regulates Nrf2, NFkB, PI3K/PKB, and p53 signal pathways to protect against oxidative stress and metabolic pathways initiating the NADPH and ATP production.

## Abbreviations

α-Syn: α-synuclein
ATP: adenosine triphosphate
Bcl-xL: B-cell lymphoma-extra large
Bcl-2: B-cell lymphoma 2
CMA: chaperone-mediated autophagy
c-Rel: proto-oncogene
DJ-1 (Park7): protein deglycase, which is encoded by the PARK7 gene
Hsc70: heat-shock cognate protein of 70 kDa
Keap1: Kelch-like ECH-associated protein 1
LAMP2A: lysosomal-associated membrane protein 2a
MMP: mitochondrial membrane potential
NADPH: nicotinamide adenine dinucleotide phosphate reduced
Nrf2: nuclear factor (erythroid-derived 2)-like 2
NQO1: NAD(P)H quinone dehydrogenase 1
6-OHDA: dopaminergic-selective neurotoxin 6-hydroxydopamine
Parkin: E3 ubiquitin ligase
PD: Parkinson's disease
PINK1: PTEN-induced putative kinase 1
PI3K/PKB: phosphatidylinositol 3-kinase/ Protein Kinase B (Akt)
PTEN: phosphatase and tensin homolog
ROS: reactive oxygen species
SNc: the substantia nigra pars compacta
SNCA: gene, which encodes protein α-synuclein
SIRT1: gene, which encodes protein NAD-dependent deacetylase sirtuin 1
UCP: mitochondrial uncoupling protein
WT: wild-type

## References

[CR1] Abeti R, Abramov AY (2015). Mitochondrial Ca(2+) in neurodegenerative disorders. Pharmacol Res.

[CR2] Abramov AY, Duchen MR (2005). The role of an astrocytic NADPH oxidase in the neurotoxicity of amyloid beta peptides. Philos Trans R Soc Lond Ser B Biol Sci.

[CR3] Abramov AY, Berezhnov AV, Fedotova EI, Zinchenko VP, Dolgacheva LP (2017) Interaction of misfolded proteins and mitochondria in neurodegenerative disorders. Biochem Soc Trans. 10.1042/BST20170024 10.1042/BST20170024 28733489

[CR4] Adams JM, Cory S (1998). The Bcl-2 protein family: arbiters of cell survival. Science.

[CR5] Adam-Vizi V, Chinopoulos C (2006). Bioenergetics and the formation of mitochondrial reactive oxygen species. Trends Pharmacol Sci.

[CR6] Alavian KN, Li H, Collis L, Bonanni L, Zeng L, Sacchetti S (2011). Bcl-xL regulates metabolic efficiency of neurons through interaction with the mitochondrial F1FO ATP synthase. Nat Cell Biol.

[CR7] Aleyasin H, Rousseaux MW, Phillips M, Kim RH, Bland RJ, Callaghan S (2007). The Parkinson's disease gene DJ-1 is also a key regulator of stroke-induced damage. Proc Natl Acad Sci U S A.

[CR8] Alvarez-Erviti L, Rodriguez-Oroz MC, Cooper JM, Caballero C, Ferrer I, Obeso JA (2010). Chaperone-mediated autophagy markers in Parkinson disease brains. Arch Neurol.

[CR9] Anderson PC, Daggett V (2008). Molecular basis for the structural instability of human DJ-1 induced by the L166P mutation associated with Parkinson's disease. Biochemistry.

[CR10] Angelova PR, Abramov AY (2016). Functional role of mitochondrial reactive oxygen species in physiology. Free Radic Biol Med.

[CR11] Annesi G, Savettieri G, Pugliese P, D'Amelio M, Tarantino P, Ragonese P (2005). DJ-1 mutations and parkinsonism-dementia-amyotrophic lateral sclerosis complex. Ann Neurol.

[CR12] Ariga H, Takahashi-Niki K, Kato I, Maita H, Niki T, Iguchi-Ariga SM (2013). Neuroprotective function of DJ-1 in Parkinson's disease. Oxidative Med Cell Longev.

[CR13] Asghar M, Banday AA, Fardoun RZ, Lokhandwala MF (2006). Hydrogen peroxide causes uncoupling of dopamine D1-like receptors from G proteins via a mechanism involving protein kinase C and G-protein-coupled receptor kinase 2. Free Radic Biol Med.

[CR14] Aumann TD, Egan K, Lim J, Boon WC, Bye CR, Chua HK (2011). Neuronal activity regulates expression of tyrosine hydroxylase in adult mouse substantia nigra pars compacta neurons. J Neurochem.

[CR15] Bader V, Ran Zhu X, Lubbert H, Stichel CC (2005). Expression of DJ-1 in the adult mouse CNS. Brain Res.

[CR16] Balaban RS, Nemoto S, Finkel T (2005). Mitochondria, oxidants, and aging. Cell.

[CR17] Bandopadhyay R, Kingsbury AE, Cookson MR, Reid AR, Evans IM, Hope AD (2004). The expression of DJ-1 (PARK7) in normal human CNS and idiopathic Parkinson's disease. Brain.

[CR18] Barzilai A, Melamed E (2003). Molecular mechanisms of selective dopaminergic neuronal death in Parkinson's disease. Trends Mol Med.

[CR19] Beckerman R, Prives C (2010). Transcriptional regulation by p53. Cold Spring Harb Perspect Biol.

[CR20] Bedard K, Krause KH (2007). The NOX family of ROS-generating NADPH oxidases: physiology and pathophysiology. Physiol Rev.

[CR21] Bejarano E, Cuervo AM (2010). Chaperone-mediated autophagy. Proc Am Thorac Soc.

[CR22] Berman SB, Chen YB, Qi B, McCaffery JM, Rucker EB, Goebbels S (2009). Bcl-x L increases mitochondrial fission, fusion, and biomass in neurons. J Cell Biol.

[CR23] Betarbet R, Sherer TB, MacKenzie G, Garcia-Osuna M, Panov AV, Greenamyre JT (2000). Chronic systemic pesticide exposure reproduces features of Parkinson's disease. Nat Neurosci.

[CR24] Bjorkblom B, Adilbayeva A, Maple-Grodem J, Piston D, Okvist M, Xu XM (2013). Parkinson disease protein DJ-1 binds metals and protects against metal-induced cytotoxicity. J Biol Chem.

[CR25] Blackinton J, Lakshminarasimhan M, Thomas KJ, Ahmad R, Greggio E, Raza AS (2009). Formation of a stabilized cysteine sulfinic acid is critical for the mitochondrial function of the parkinsonism protein DJ-1. J Biol Chem.

[CR26] Bonifati V (2012). Autosomal recessive parkinsonism. Parkinsonism Relat Disord.

[CR27] Bonifati V, Rizzu P, Squitieri F, Krieger E, Vanacore N, van Swieten JC (2003). DJ-1( PARK7), a novel gene for autosomal recessive, early onset parkinsonism. Neurol Sci.

[CR28] Bonifati V, Rizzu P, van Baren MJ, Schaap O, Breedveld GJ, Krieger E (2003). Mutations in the DJ-1 gene associated with autosomal recessive early-onset parkinsonism. Science.

[CR29] Braak H, Sandmann-Keil D, Gai W, Braak E (1999). Extensive axonal Lewy neurites in Parkinson's disease: a novel pathological feature revealed by alpha-synuclein immunocytochemistry. Neurosci Lett.

[CR30] Braak H, Ghebremedhin E, Rub U, Bratzke H, Del Tredici K (2004). Stages in the development of Parkinson's disease-related pathology. Cell Tissue Res.

[CR31] Bretaud S, Allen C, Ingham PW, Bandmann O (2007). p53-dependent neuronal cell death in a DJ-1-deficient zebrafish model of Parkinson's disease. J Neurochem.

[CR32] Canet-Aviles RM, Wilson MA, Miller DW, Ahmad R, McLendon C, Bandyopadhyay S (2004). The Parkinson's disease protein DJ-1 is neuroprotective due to cysteine-sulfinic acid-driven mitochondrial localization. Proc Natl Acad Sci U S A.

[CR33] Cao J, Lou S, Ying M, Yang B (2015). DJ-1 as a human oncogene and potential therapeutic target. Biochem Pharmacol.

[CR34] Chan CS, Guzman JN, Ilijic E, Mercer JN, Rick C, Tkatch T (2007). Rejuvenation' protects neurons in mouse models of Parkinson's disease. Nature.

[CR35] Chartier-Harlin MC, Kachergus J, Roumier C, Mouroux V, Douay X, Lincoln S (2004). Alpha-synuclein locus duplication as a cause of familial Parkinson's disease. Lancet.

[CR36] Chen J, Li L, Chin LS (2010). Parkinson disease protein DJ-1 converts from a zymogen to a protease by carboxyl-terminal cleavage. Hum Mol Genet.

[CR37] Chen YB, Aon MA, Hsu YT, Soane L, Teng X, McCaffery JM (2011). Bcl-xL regulates mitochondrial energetics by stabilizing the inner membrane potential. J Cell Biol.

[CR38] Cheng EH, Levine B, Boise LH, Thompson CB, Hardwick JM (1996). Bax-independent inhibition of apoptosis by Bcl-XL. Nature.

[CR39] Chien WL, Lee TR, Hung SY, Kang KH, Wu RM, Lee MJ (2013). Increase of oxidative stress by a novel PINK1 mutation, P209A. Free Radic Biol Med.

[CR40] Choi J, Sullards MC, Olzmann JA, Rees HD, Weintraub ST, Bostwick DE (2006). Oxidative damage of DJ-1 is linked to sporadic Parkinson and Alzheimer diseases. J Biol Chem.

[CR41] Choi MS, Nakamura T, Cho SJ, Han X, Holland EA, Qu J (2014). Transnitrosylation from DJ-1 to PTEN attenuates neuronal cell death in parkinson's disease models. J Neurosci.

[CR42] Clements CM, McNally RS, Conti BJ, Mak TW, Ting JP (2006). DJ-1, a cancer- and Parkinson's disease-associated protein, stabilizes the antioxidant transcriptional master regulator Nrf2. Proc Natl Acad Sci U S A.

[CR43] Cookson MR (2003). Pathways to Parkinsonism. Neuron.

[CR44] Cuervo AM, Wong E (2014). Chaperone-mediated autophagy: roles in disease and aging. Cell Res.

[CR45] Cuervo AM, Stefanis L, Fredenburg R, Lansbury PT, Sulzer D (2004). Impaired degradation of mutant alpha-synuclein by chaperone-mediated autophagy. Science.

[CR46] Denton RM (2009). Regulation of mitochondrial dehydrogenases by calcium ions. Biochim Biophys Acta.

[CR47] Dice JF (2007). Chaperone-mediated autophagy. Autophagy.

[CR48] Dinkova-Kostova AT, Baird L, Holmstrom KM, Meyer CJ, Abramov AY (2015). The spatiotemporal regulation of the Keap1-Nrf2 pathway and its importance in cellular bioenergetics. Biochem Soc Trans.

[CR49] Dohi K, Ohtaki H, Nakamachi T, Yofu S, Satoh K, Miyamoto K (2010). Gp91phox (NOX2) in classically activated microglia exacerbates traumatic brain injury. J Neuroinflammation.

[CR50] Droge W (2002). Free radicals in the physiological control of cell function. Physiol Rev.

[CR51] Duplan E, Giaime E, Viotti J, Sevalle J, Corti O, Brice A (2013). ER-stress-associated functional link between Parkin and DJ-1 via a transcriptional cascade involving the tumor suppressor p53 and the spliced X-box binding protein XBP-1. J Cell Sci.

[CR52] Duplan E, Giordano C, Checler F, Alves da Costa C (2016). Direct alpha-synuclein promoter transactivation by the tumor suppressor p53. Mol Neurodegener.

[CR53] el-Deiry WS, Kern SE, Pietenpol JA, Kinzler KW, Vogelstein B (1992). Definition of a consensus binding site for p53. Nat Genet.

[CR54] Esteras N, Dinkova-Kostova AT, Abramov AY (2016). Nrf2 activation in the treatment of neurodegenerative diseases: a focus on its role in mitochondrial bioenergetics and function. Biol Chem.

[CR55] Fahn S (2003). Description of Parkinson's disease as a clinical syndrome. Ann N Y Acad Sci.

[CR56] Fan J, Ren H, Fei E, Jia N, Ying Z, Jiang P (2008). Sumoylation is critical for DJ-1 to repress p53 transcriptional activity. FEBS Lett.

[CR57] Fan J, Yu H, Lv Y, Yin L (2016). Diagnostic and prognostic value of serum thioredoxin and DJ-1 in non-small cell lung carcinoma patients. Tumour Biol.

[CR58] Fardoun RZ, Asghar M, Lokhandwala M (2007). Role of nuclear factor kappa B (NF-kappaB) in oxidative stress-induced defective dopamine D1 receptor signaling in the renal proximal tubules of Sprague-Dawley rats. Free Radic Biol Med.

[CR59] Fu Y, Paxinos G, Watson C, Halliday GM (2016). The substantia nigra and ventral tegmental dopaminergic neurons from development to degeneration. J Chem Neuroanat.

[CR60] Funk WD, Pak DT, Karas RH, Wright WE, Shay JW (1992). A transcriptionally active DNA-binding site for human p53 protein complexes. Mol Cell Biol.

[CR61] Gan L, Johnson DA, Johnson JA (2010). Keap1-Nrf2 activation in the presence and absence of DJ-1. Eur J Neurosci.

[CR62] Gandhi S, Abramov AY (2012). Mechanism of oxidative stress in neurodegeneration. Oxidative Med Cell Longev.

[CR63] Gellerich FN, Gizatullina Z, Arandarcikaite O, Jerzembek D, Vielhaber S, Seppet E (2009). Extramitochondrial Ca2+ in the nanomolar range regulates glutamate-dependent oxidative phosphorylation on demand. PLoS One.

[CR64] Ghosh D, LeVault KR, Brewer GJ (2014). Dual-energy precursor and nuclear erythroid-related factor 2 activator treatment additively improve redox glutathione levels and neuron survival in aging and Alzheimer mouse neurons upstream of reactive oxygen species. Neurobiol Aging.

[CR65] Giaime E, Sunyach C, Druon C, Scarzello S, Robert G, Grosso S (2010). Loss of function of DJ-1 triggered by Parkinson's disease-associated mutation is due to proteolytic resistance to caspase-6. Cell Death Differ.

[CR66] Gorrini C, Baniasadi PS, Harris IS, Silvester J, Inoue S, Snow B (2013). BRCA1 interacts with Nrf2 to regulate antioxidant signaling and cell survival. J Exp Med.

[CR67] Grace AA, Bunney BS (1983). Intracellular and extracellular electrophysiology of nigral dopaminergic neurons--1. Identification and characterization. Neuroscience.

[CR68] Grace AA, Bunney BS (1983). Intracellular and extracellular electrophysiology of nigral dopaminergic neurons--2. Action potential generating mechanisms and morphological correlates. Neuroscience.

[CR69] Gu B, Zhu WG (2012). Surf the post-translational modification network of p53 regulation. Int J Biol Sci.

[CR70] Gunjima K, Tomiyama R, Takakura K, Yamada T, Hashida K, Nakamura Y (2014). 3,4-dihydroxybenzalacetone protects against Parkinson's disease-related neurotoxin 6-OHDA through Akt/Nrf2/glutathione pathway. J Cell Biochem.

[CR71] Guzman JN, Sanchez-Padilla J, Chan CS, Surmeier DJ (2009). Robust pacemaking in substantia nigra dopaminergic neurons. J Neurosci.

[CR72] Guzman JN, Sanchez-Padilla J, Wokosin D, Kondapalli J, Ilijic E, Schumacker PT (2010). Oxidant stress evoked by pacemaking in dopaminergic neurons is attenuated by DJ-1. Nature.

[CR73] Hayashi T, Ishimori C, Takahashi-Niki K, Taira T, Kim YC, Maita H (2009). DJ-1 binds to mitochondrial complex I and maintains its activity. Biochem Biophys Res Commun.

[CR74] Henley JM, Carmichael RE, Wilkinson KA (2018). Extranuclear SUMOylation in Neurons. Trends Neurosci.

[CR75] Ho JW, Ho PW, Zhang WY, Liu HF, Kwok KH, Yiu DC (2010). Transcriptional regulation of UCP4 by NF-kappaB and its role in mediating protection against MPP+ toxicity. Free Radic Biol Med.

[CR76] Ho JW, Ho PW, Liu HF, So DH, Chan KH, Tse ZH (2012). UCP4 is a target effector of the NF-kappaB c-Rel prosurvival pathway against oxidative stress. Free Radic Biol Med.

[CR77] Ho PW, Ho JW, Liu HF, So DH, Tse ZH, Chan KH (2012). Mitochondrial neuronal uncoupling proteins: a target for potential disease-modification in Parkinson's disease. Transl Neurodegener.

[CR78] Hoffmann A, Baltimore D (2006). Circuitry of nuclear factor kappaB signaling. Immunol Rev.

[CR79] Holmstrom KM, Marina N, Baev AY, Wood NW, Gourine AV, Abramov AY (2013). Signalling properties of inorganic polyphosphate in the mammalian brain. Nat Commun.

[CR80] Honbou K, Suzuki NN, Horiuchi M, Niki T, Taira T, Ariga H (2003). The crystal structure of DJ-1, a protein related to male fertility and Parkinson's disease. J Biol Chem.

[CR81] Horn HF, Vousden KH (2007). Coping with stress: multiple ways to activate p53. Oncogene.

[CR82] Huai Q, Sun Y, Wang H, Chin LS, Li L, Robinson H (2003). Crystal structure of DJ-1/RS and implication on familial Parkinson's disease. FEBS Lett.

[CR83] Im JY, Lee KW, Junn E, Mouradian MM (2010). DJ-1 protects against oxidative damage by regulating the thioredoxin/ASK1 complex. Neurosci Res.

[CR84] Im JY, Lee KW, Woo JM, Junn E, Mouradian MM (2012). DJ-1 induces thioredoxin 1 expression through the Nrf2 pathway. Hum Mol Genet.

[CR85] Inberg A, Linial M (2010). Protection of pancreatic beta-cells from various stress conditions is mediated by DJ-1. J Biol Chem.

[CR86] Inden M, Taira T, Kitamura Y, Yanagida T, Tsuchiya D, Takata K (2006). PARK7 DJ-1 protects against degeneration of nigral dopaminergic neurons in Parkinson's disease rat model. Neurobiol Dis.

[CR87] Inden M, Kitamura Y, Takahashi K, Takata K, Ito N, Niwa R (2011). Protection against dopaminergic neurodegeneration in Parkinson's disease-model animals by a modulator of the oxidized form of DJ-1, a wild-type of familial Parkinson's disease-linked PARK7. J Pharmacol Sci.

[CR88] Irrcher I, Aleyasin H, Seifert EL, Hewitt SJ, Chhabra S, Phillips M (2010). Loss of the Parkinson's disease-linked gene DJ-1 perturbs mitochondrial dynamics. Hum Mol Genet.

[CR89] Ito G, Ariga H, Nakagawa Y, Iwatsubo T (2006). Roles of distinct cysteine residues in S-nitrosylation and dimerization of DJ-1. Biochem Biophys Res Commun.

[CR90] Jain D, Jain R, Eberhard D, Eglinger J, Bugliani M, Piemonti L (2012). Age- and diet-dependent requirement of DJ-1 for glucose homeostasis in mice with implications for human type 2 diabetes. J Mol Cell Biol.

[CR91] Jenner P (2003). The MPTP-treated primate as a model of motor complications in PD: primate model of motor complications. Neurology.

[CR92] Junn E, Jang WH, Zhao X, Jeong BS, Mouradian MM (2009). Mitochondrial localization of DJ-1 leads to enhanced neuroprotection. J Neurosci Res.

[CR93] Kahle PJ, Waak J, Gasser T (2009). DJ-1 and prevention of oxidative stress in Parkinson's disease and other age-related disorders. Free Radic Biol Med.

[CR94] Kang S, Cooper G, Dunne SF, Dusel B, Luan CH, Surmeier DJ (2012). CaV1.3-selective L-type calcium channel antagonists as potential new therapeutics for Parkinson's disease. Nat Commun.

[CR95] Kato I, Maita H, Takahashi-Niki K, Saito Y, Noguchi N, Iguchi-Ariga SM (2013). Oxidized DJ-1 inhibits p53 by sequestering p53 from promoters in a DNA-binding affinity-dependent manner. Mol Cell Biol.

[CR96] Kaushik S, Cuervo AM (2009). Methods to monitor chaperone-mediated autophagy. Methods Enzymol.

[CR97] Kensler TW, Wakabayashi N, Biswal S (2007). Cell survival responses to environmental stresses via the Keap1-Nrf2-ARE pathway. Annu Rev Pharmacol Toxicol.

[CR98] Kharbanda S, Pandey P, Schofield L, Israels S, Roncinske R, Yoshida K (1997). Role for Bcl-xL as an inhibitor of cytosolic cytochrome C accumulation in DNA damage-induced apoptosis. Proc Natl Acad Sci U S A.

[CR99] Kim SJ, Park YJ, Hwang IY, Youdim MB, Park KS, Oh YJ (2012). Nuclear translocation of DJ-1 during oxidative stress-induced neuronal cell death. Free Radic Biol Med.

[CR100] Kinumi T, Kimata J, Taira T, Ariga H, Niki E (2004). Cysteine-106 of DJ-1 is the most sensitive cysteine residue to hydrogen peroxide-mediated oxidation in vivo in human umbilical vein endothelial cells. Biochem Biophys Res Commun.

[CR101] Kovac S, Angelova PR, Holmstrom KM, Zhang Y, Dinkova-Kostova AT, Abramov AY (2015). Nrf2 regulates ROS production by mitochondria and NADPH oxidase. Biochim Biophys Acta.

[CR102] Krebiehl G, Ruckerbauer S, Burbulla LF, Kieper N, Maurer B, Waak J (2010). Reduced basal autophagy and impaired mitochondrial dynamics due to loss of Parkinson's disease-associated protein DJ-1. PLoS One.

[CR103] Kretz-Remy C, Mehlen P, Mirault ME, Arrigo AP (1996). Inhibition of I kappa B-alpha phosphorylation and degradation and subsequent NF-kappa B activation by glutathione peroxidase overexpression. J Cell Biol.

[CR104] Kruger R, Kuhn W, Muller T, Woitalla D, Graeber M, Kosel S (1998). Ala30Pro mutation in the gene encoding alpha-synuclein in Parkinson's disease. Nat Genet.

[CR105] Lee SJ, Kim SJ, Kim IK, Ko J, Jeong CS, Kim GH (2003). Crystal structures of human DJ-1 and Escherichia coli Hsp31, which share an evolutionarily conserved domain. J Biol Chem.

[CR106] Lee H, Choi SK, Ro JY (2012). Overexpression of DJ-1 and HSP90 alpha, and loss of PTEN associated with invasive urothelial carcinoma of urinary bladder: Possible prognostic markers. Oncol Lett.

[CR107] Lee MK, Lee MS, Bae DW, Lee DH, Cha SS, Chi SW (2018). Structural basis for the interaction between DJ-1 and Bcl-XL. Biochem Biophys Res Commun.

[CR108] Li C, Wang X, Vais H, Thompson CB, Foskett JK, White C (2007). Apoptosis regulation by Bcl-x(L) modulation of mammalian inositol 1,4,5-trisphosphate receptor channel isoform gating. Proc Natl Acad Sci U S A.

[CR109] Liu F, Nguyen JL, Hulleman JD, Li L, Rochet JC (2008). Mechanisms of DJ-1 neuroprotection in a cellular model of Parkinson's disease. J Neurochem.

[CR110] Ludtmann MHR, Abramov AY (2018). Mitochondrial calcium imbalance in Parkinson's disease. Neurosci Lett.

[CR111] Majeski AE, Dice JF (2004). Mechanisms of chaperone-mediated autophagy. Int J Biochem Cell Biol.

[CR112] Mandemakers W, Morais VA, De Strooper B (2007). A cell biological perspective on mitochondrial dysfunction in Parkinson disease and other neurodegenerative diseases. J Cell Sci.

[CR113] Martinat C, Shendelman S, Jonason A, Leete T, Beal MF, Yang L (2004). Sensitivity to oxidative stress in DJ-1-deficient dopamine neurons: an ES- derived cell model of primary Parkinsonism. PLoS Biol.

[CR114] Meulener MC, Graves CL, Sampathu DM, Armstrong-Gold CE, Bonini NM, Giasson BI (2005). DJ-1 is present in a large molecular complex in human brain tissue and interacts with alpha-synuclein. J Neurochem.

[CR115] Mitsumoto A, Nakagawa Y (2001). DJ-1 is an indicator for endogenous reactive oxygen species elicited by endotoxin. Free Radic Res.

[CR116] Moore DJ, Zhang L, Dawson TM, Dawson VL (2003). A missense mutation (L166P) in DJ-1, linked to familial Parkinson's disease, confers reduced protein stability and impairs homo-oligomerization. J Neurochem.

[CR117] Moore DJ, Zhang L, Troncoso J, Lee MK, Hattori N, Mizuno Y (2005). Association of DJ-1 and parkin mediated by pathogenic DJ-1 mutations and oxidative stress. Hum Mol Genet.

[CR118] Moore DJ, Dawson VL, Dawson TM (2006). Lessons from Drosophila models of DJ-1 deficiency. Sci Aging Knowl Environ.

[CR119] Moors T, Paciotti S, Chiasserini D, Calabresi P, Parnetti L, Beccari T (2016). Lysosomal Dysfunction and alpha-Synuclein Aggregation in Parkinson's Disease: Diagnostic Links. Mov Disord.

[CR120] Moscovitz O, Ben-Nissan G, Fainer I, Pollack D, Mizrachi L, Sharon M (2015). The Parkinson's-associated protein DJ-1 regulates the 20S proteasome. Nat Commun.

[CR121] Motohashi H, Yamamoto M (2004). Nrf2-Keap1 defines a physiologically important stress response mechanism. Trends Mol Med.

[CR122] Mullett SJ, Di Maio R, Greenamyre JT, Hinkle DA (2013). DJ-1 expression modulates astrocyte-mediated protection against neuronal oxidative stress. J Mol Neurosci.

[CR123] Murphy KE, Gysbers AM, Abbott SK, Spiro AS, Furuta A, Cooper A (2015). Lysosomal-associated membrane protein 2 isoforms are differentially affected in early Parkinson's disease. Mov Disord.

[CR124] Nagakubo D, Taira T, Kitaura H, Ikeda M, Tamai K, Iguchi-Ariga SM (1997). DJ-1, a novel oncogene which transforms mouse NIH3T3 cells in cooperation with ras. Biochem Biophys Res Commun.

[CR125] Numajiri N, Takasawa K, Nishiya T, Tanaka H, Ohno K, Hayakawa W (2011). On-off system for PI3-kinase-Akt signaling through S-nitrosylation of phosphatase with sequence homology to tensin (PTEN). Proc Natl Acad Sci U S A.

[CR126] Pacelli C, Giguere N, Bourque MJ, Levesque M, Slack RS, Trudeau LE (2015). Elevated Mitochondrial Bioenergetics and Axonal Arborization Size Are Key Contributors to the Vulnerability of Dopamine Neurons. Curr Biol.

[CR127] Park L, Zhou P, Pitstick R, Capone C, Anrather J, Norris EH (2008). Nox2-derived radicals contribute to neurovascular and behavioral dysfunction in mice overexpressing the amyloid precursor protein. Proc Natl Acad Sci U S A.

[CR128] Parsanejad M, Bourquard N, Qu D, Zhang Y, Huang E, Rousseaux MW (2014). DJ-1 interacts with and regulates paraoxonase-2, an enzyme critical for neuronal survival in response to oxidative stress. PLoS One.

[CR129] Pemberton S, Madiona K, Pieri L, Kabani M, Bousset L, Melki R (2011). Hsc70 protein interaction with soluble and fibrillar alpha-synuclein. J Biol Chem.

[CR130] Petros AM, Nettesheim DG, Wang Y, Olejniczak ET, Meadows RP, Mack J (2000). Rationale for Bcl-xL/Bad peptide complex formation from structure, mutagenesis, and biophysical studies. Protein Sci.

[CR131] Petry A, Weitnauer M, Gorlach A (2010). Receptor activation of NADPH oxidases. Antioxid Redox Signal.

[CR132] Piston D, Alvarez-Erviti L, Bansal V, Gargano D, Yao Z, Szabadkai G (2017). DJ-1 is a redox sensitive adapter protein for high molecular weight complexes involved in regulation of catecholamine homeostasis. Hum Mol Genet.

[CR133] Polymeropoulos MH, Lavedan C, Leroy E, Ide SE, Dehejia A, Dutra A (1997). Mutation in the alpha-synuclein gene identified in families with Parkinson's disease. Science.

[CR134] Przedborski S, Tieu K, Perier C, Vila M (2004). MPTP as a mitochondrial neurotoxic model of Parkinson's disease. J Bioenerg Biomembr.

[CR135] Rajendra R, Malegaonkar D, Pungaliya P, Marshall H, Rasheed Z, Brownell J (2004). Topors functions as an E3 ubiquitin ligase with specific E2 enzymes and ubiquitinates p53. J Biol Chem.

[CR136] Ramsden DB, Ho PW, Ho JW, Liu HF, So DH, Tse HM (2012). Human neuronal uncoupling proteins 4 and 5 (UCP4 and UCP5): structural properties, regulation, and physiological role in protection against oxidative stress and mitochondrial dysfunction. Brain Behav.

[CR137] Raninga PV, Di Trapani G, Tonissen KF (2017). The Multifaceted Roles of DJ-1 as an Antioxidant. Adv Exp Med Biol.

[CR138] Ren H, Fu K, Wang D, Mu C, Wang G (2011). Oxidized DJ-1 interacts with the mitochondrial protein BCL-XL. J Biol Chem.

[CR139] Ren H, Fu K, Mu C, Zhen X, Wang G (2012). L166P mutant DJ-1 promotes cell death by dissociating Bax from mitochondrial Bcl-XL. Mol Neurodegener.

[CR140] Richarme G, Dairou J (2017). Parkinsonism-associated protein DJ-1 is a bona fide deglycase. Biochem Biophys Res Commun.

[CR141] Sattler M, Liang H, Nettesheim D, Meadows RP, Harlan JE, Eberstadt M (1997). Structure of Bcl-xL-Bak peptide complex: recognition between regulators of apoptosis. Science.

[CR142] Schapira AH, Jenner P (2011). Etiology and pathogenesis of Parkinson's disease. Mov Disord.

[CR143] Schreck R, Baeuerle PA (1991). A role for oxygen radicals as second messengers. Trends Cell Biol.

[CR144] Schreck R, Rieber P, Baeuerle PA (1991). Reactive oxygen intermediates as apparently widely used messengers in the activation of the NF-kappa B transcription factor and HIV-1. EMBO J.

[CR145] Seppet EK, Eimre M, Andrienko T, Kaambre T, Sikk P, Kuznetsov AV (2004). Studies of mitochondrial respiration in muscle cells in situ: use and misuse of experimental evidence in mathematical modelling. Mol Cell Biochem.

[CR146] Shendelman S, Jonason A, Martinat C, Leete T, Abeliovich A (2004). DJ-1 is a redox-dependent molecular chaperone that inhibits alpha-synuclein aggregate formation. PLoS Biol.

[CR147] Sherer, T. B., Betarbet, R., Stout, A. K., Lund, S., Baptista, M., Panov, A. V., et al. (2002). An in vitro model of Parkinson's disease: linking mitochondrial impairment to altered alpha-synuclein metabolism and oxidative damage. J Neurosci, 22(16), 7006-7015 10.1523/JNEUROSCI.22-16-07006.2002 PMC6757862 12177198

[CR148] Shinbo Y, Taira T, Niki T, Iguchi-Ariga SM, Ariga H (2005). DJ-1 restores p53 transcription activity inhibited by Topors/p53BP3. Int J Oncol.

[CR149] Simon-Sanchez J, Schulte C, Bras JM, Sharma M, Gibbs JR, Berg D (2009). Genome-wide association study reveals genetic risk underlying Parkinson's disease. Nat Genet.

[CR150] Starkov AA (2008). The role of mitochondria in reactive oxygen species metabolism and signaling. Ann N Y Acad Sci.

[CR151] Surmeier DJ, Schumacker PT (2013). Calcium, bioenergetics, and neuronal vulnerability in Parkinson's disease. J Biol Chem.

[CR152] Surmeier DJ, Guzman JN, Sanchez-Padilla J, Goldberg JA (2010). What causes the death of dopaminergic neurons in Parkinson's disease?. Prog Brain Res.

[CR153] Surmeier DJ, Halliday GM, Simuni T (2017). Calcium, mitochondrial dysfunction and slowing the progression of Parkinson's disease. Exp Neurol.

[CR154] Taira T, Saito Y, Niki T, Iguchi-Ariga SM, Takahashi K, Ariga H (2004). DJ-1 has a role in antioxidative stress to prevent cell death. EMBO Rep.

[CR155] Takahashi-Niki K, Ganaha Y, Niki T, Nakagawa S, Kato-Ose I, Iguchi-Ariga SMM (2016). DJ-1 activates SIRT1 through its direct binding to SIRT1. Biochem Biophys Res Commun.

[CR156] Tanti GK, Goswami SK (2014). SG2NA recruits DJ-1 and Akt into the mitochondria and membrane to protect cells from oxidative damage. Free Radic Biol Med.

[CR157] Tao X, Tong L (2003). Crystal structure of human DJ-1, a protein associated with early onset Parkinson's disease. J Biol Chem.

[CR158] Thomas KJ, McCoy MK, Blackinton J, Beilina A, van der Brug M, Sandebring A (2011). DJ-1 acts in parallel to the PINK1/parkin pathway to control mitochondrial function and autophagy. Hum Mol Genet.

[CR159] Trempe JF, Fon EA (2013). Structure and Function of Parkin, PINK1, and DJ-1, the Three Musketeers of Neuroprotection. Front Neurol.

[CR160] Trojanowski JQ, Lee VM (1998). Aggregation of neurofilament and alpha-synuclein proteins in Lewy bodies: implications for the pathogenesis of Parkinson disease and Lewy body dementia. Arch Neurol.

[CR161] Valente EM, Abou-Sleiman PM, Caputo V, Muqit MM, Harvey K, Gispert S (2004). Hereditary early-onset Parkinson's disease caused by mutations in PINK1. Science.

[CR162] van Horssen J, Drexhage JA, Flor T, Gerritsen W, van der Valk P, de Vries HE (2010). Nrf2 and DJ1 are consistently upregulated in inflammatory multiple sclerosis lesions. Free Radic Biol Med.

[CR163] Velseboer DC, de Haan RJ, Wieling W, Goldstein DS, de Bie RM (2011). Prevalence of orthostatic hypotension in Parkinson's disease: a systematic review and meta-analysis. Parkinsonism Relat Disord.

[CR164] Vogiatzi T, Xilouri M, Vekrellis K, Stefanis L (2008). Wild type alpha-synuclein is degraded by chaperone-mediated autophagy and macroautophagy in neuronal cells. J Biol Chem.

[CR165] Wilson MA (2011). The role of cysteine oxidation in DJ-1 function and dysfunction. Antioxid Redox Signal.

[CR166] Wilson MA, Collins JL, Hod Y, Ringe D, Petsko GA (2003). The 1.1-A resolution crystal structure of DJ-1, the protein mutated in autosomal recessive early onset Parkinson's disease. Proc Natl Acad Sci U S A.

[CR167] Xilouri M, Brekk OR, Landeck N, Pitychoutis PM, Papasilekas T, Papadopoulou-Daifoti Z (2013). Boosting chaperone-mediated autophagy in vivo mitigates alpha-synuclein-induced neurodegeneration. Brain.

[CR168] Xu CY, Kang WY, Chen YM, Jiang TF, Zhang J, Zhang LN (2017). DJ-1 Inhibits alpha-Synuclein Aggregation by Regulating Chaperone-Mediated Autophagy. Front Aging Neurosci.

[CR169] Xu S, Yang X, Qian Y, Xiao Q (2018) Parkinson disease-related DJ-1 modulates the expression of uncoupling protein 4 against oxidative stress. J Neurochem. 10.1111/jnc.14297 10.1111/jnc.14297 29315581

[CR170] Yamaguchi S, Yamane T, Takahashi-Niki K, Kato I, Niki T, Goldberg MS (2012). Transcriptional activation of low-density lipoprotein receptor gene by DJ-1 and effect of DJ-1 on cholesterol homeostasis. PLoS One.

[CR171] Yang Q, She H, Gearing M, Colla E, Lee M, Shacka JJ (2009). Regulation of neuronal survival factor MEF2D by chaperone-mediated autophagy. Science.

[CR172] Yu D, Pan HX, Zhang RG, Li Y, Nie X (2017). Nucleus DJ-1/Park7 acts as a favorable prognostic factor and involves mucin secretion in invasive breast carcinoma in Chinese population. Int J Clin Exp Med.

[CR173] Zaltieri M, Longhena F, Pizzi M, Missale C, Spano P, Bellucci A (2015). Mitochondrial Dysfunction and alpha-Synuclein Synaptic Pathology in Parkinson's Disease: Who's on First?. Parkinsons Dis.

[CR174] Zarranz JJ, Alegre J, Gomez-Esteban JC, Lezcano E, Ros R, Ampuero I (2004). The new mutation, E46K, of alpha-synuclein causes Parkinson and Lewy body dementia. Ann Neurol.

[CR175] Zhang L, Shimoji M, Thomas B, Moore DJ, Yu SW, Marupudi NI (2005). Mitochondrial localization of the Parkinson's disease related protein DJ-1: implications for pathogenesis. Hum Mol Genet.

[CR176] Zhou W, Freed CR (2005). DJ-1 up-regulates glutathione synthesis during oxidative stress and inhibits A53T alpha-synuclein toxicity. J Biol Chem.

[CR177] Zhou W, Zhu M, Wilson MA, Petsko GA, Fink AL (2006). The oxidation state of DJ-1 regulates its chaperone activity toward alpha-synuclein. J Mol Biol.

[CR178] Zhu JH, Chu CT (2010). Mitochondrial Dysfunction in Parkinson's Disease. J Alzheimers Dis.

[CR179] Zondler L, Miller-Fleming L, Repici M, Goncalves S, Tenreiro S, Rosado-Ramos R (2014). DJ-1 interactions with alpha-synuclein attenuate aggregation and cellular toxicity in models of Parkinson's disease. Cell Death Dis.

